# Accelerating HIV and AIDS services delivery in Kigoma region, Tanzania

**DOI:** 10.11604/pamj.supp.2023.45.1.39597

**Published:** 2023-07-10

**Authors:** Jairos Hiliza, Ernest Ndizeimana, Hosea William, Jesca Lebba, Christine Musanhu, Peter Nsubuga, Yoti Zablon

**Affiliations:** 1 World Health Organization, Dar es Salaam, Tanzania,; 2 Kigoma Health Management Team, Kigoma, Tanzania,; 3 Global public health solutions, Dar es Salaam, Tanzania

**Keywords:** HIV and AIDS, service delivery, Kigoma, Tanzania

## Abstract

**Introduction:**

Tanzania Commission for AIDS and UNAIDS reports 1.7 million Tanzanians are HIV-positive. The Joint United Nations Program on HIV/AIDS set 95%, 95% 95% targets to be achieved by 2025. An assessment was done to understand the region’s position, which found the underperformance of critical HIV and AIDS indicators. This prompted the region to accelerate HIV interventions by providing frontline healthcare providers with skills and knowledge, essential equipment, and other infrastructure, after which the assessment of the indicators was conducted to document the outcome of interventions.

**Methods:**

we conducted a descriptive study in Kigoma region in June 2022 by comparing HIV and AIDS indicators performance in the pre-intervention and post interventions arms. High-volume CTCs were purposefully selected. We used a pre-tested checklist to assess new HIV-positive on antiretroviral (ARV), pregnant women living with HIV on ARV, and people living with HIV offered multi-month dispensing. We further assessed HIV viral load (HVL) specimen collection, HIV suppression status, and HVL test results turnaround time. We cleaned the information using an MS Excel sheet and tabulated results using STATA software version 13.

**Results:**

we assessed 27 care and clinics. The proportion gain in the index client elicitation was 13%. Sexual partners mentioned during index client elicitation had an increase of 145 individuals. The yield among consented sexual partners gained by 14%. The ARV initiation among new HIV -positive and pregnant women living with HIV gained a proportion of 2%. Multi-month dispencing was found to have an 8% increase. The turnaround time for HVL test results decreased by 21 days, and the viral load suppression status increased by 4%.

**Conclusion:**

the assessment demonstrated the accelerated HIV and AIDS service delivery due to implementing a comprehensive package of HIV and AIDS management. We recommend in-service capacity building regarding training, basic equipment, and infrastructure.

## Introduction

The strategies and mitigation of the United Nations (UN) to end HIV and AIDS by 2030 have been set as part of the Sustainable Development Goals [1]. In December 2020, the Joint United Nations Program on HIV/AIDS (UNAIDS) set new targets to be achieved by 2025. The target is to ensure 95% of people living with HIV and AIDS (PLHIV) know their status, 95% of all people who tested HIV positive are receiving antiretroviral treatment (ART), and 95% of all people who are receiving ART are virally suppressed [2].

Records from the Ministry of Health, Tanzania Commission for AIDS (TACIDS), and UNAIDS indicate that about 1.7 million, or 4.8% of Tanzanians aged 15-49, are HIV-positive with approximately 77,000 new HIV infections and 27,000 AIDS-related deaths annually [3]. According to Tanzania HIV Impact Survey, which was conducted in 2017, The HIV prevalence in Kigoma region was estimated to be 2.9%. During this survey study, Kigoma’s prevalence rate was low compared to the other nearby regions of Tabora, Geita, and Katavi [3]. Literature shows that the new HIV infections and transmission of HIV in Kigoma are prevalent among refugee populations who are enclosed in the camps with no physical activities [4]. HIV is also prevalent among the migratory fishermen along Lake Tanganyika shore who work and stay away from their families for a long time [5]. The availability of a high number of roaming cattle-rearing communities who usually move from high-burden HIV regions to Kigoma region has increased the HIV burden in Kigoma Region [6,7].

Skilled and knowledgeable healthcare providers have been shown to accelerate the decline of HIV and AIDS morbidity, mortality, and its sequelae [8,9]. According to the assessment conducted at Kigoma HIV care and treatment clinics in January-February 2022, some indicators that could lead to the achievement of the 95 95 95 targets by 2025 were underperforming. The DHIS2 data show that testing coverage declined by 17% in 2021 compared to 2020. Moreover, health facilities were also notably struggling to provide services for ART initiation, multi months dispensing (MMD), and HIV viral load (HVL) coverage.

Kigoma region decided to comprehensively accelerate the HIV interventions, including providing frontline healthcare providers with skills and knowledge, provision of essential equipment, and other infrastructure per National AIDS control guidelines to institutional capacity to provide HIV and AIDS care to PLHIV [10]. Afterward, the region assessed the HIV and AIDS service delivery. The study aimed at describing the changes in HIV and AIDS service delivery indicators following the implementation of comprehensive HIV and AIDS management interventions.

## Methods

**Study setting:** Kigoma region is in the northwestern corner of Tanzania, on the eastern shore of Lake Tanganyika. It borders the Democratic Republic of Congo to the west through Lake Tanganyika, the Republic of Burundi to the North-West, Kagera region to the North, the region Borders Tabora and Geita region to the East, and Katavi region to the South. According to the Tanzania National Census conducted in 2022, the region has a population of 2.5 million (1.2 million males and 1.3 million females). The region has 286 health facilities, including nine hospitals, 37 health centers, and 240 dispensaries. Of the 286 health facilities, 83 provide care and treatment services to people living with HIV, and 183 are standalone facilities that provide Prevention of MotherTo Child Transmission (PMTCT) services. The region has several health implementing partners, including Tanzania Health Promotion Support (THPS) which provides HIV and AIDS support to 62 (22%) of health facilities in the region, and Management and Development for Health (MDH), which focus on voluntary medical male circumcision (VMMC). In the region, there are other vertical programs from the Ministry of Health, operating under the National AIDS Control Program (NACP) and Tanzania Commission for AIDS (TACAIDS). The vertical programs cover the provision of condoms, antiretroviral drugs, HIV rapid testing kits, polymerase chain reaction (PCR) machines, and service provision registers.

**Intervention implemented:** we designed and implemented a comprehensive campaign to ensure all care and treatment clinics (CTCs) and standalone Prevention of Mother to Child Transmission (PMTCT) clinics have all the necessary equipment and skills. We assessed the availability of rapid diagnostic kits for HIV, antiretroviral (ARV) drugs (adult and pediatric formulations), HIV viral load and dried blood spot (DBS) collection kits, working PCR machines, and paper or computerized data recording systems. In the CTCs and PMTCT standalone facilities, which had deficits, we refilled the missing items to meet the NACP prerequisites. Then, from each CTCs and PMTCT standalone health facility, we comprehensively trained healthcare providers and peer educators on providing HIV and AIDS services, including health facilities and community-based HIV testing strategies, ARV dispensing, client enrollment, client tracking and tracing back, and index client testing. We further trained healthcare providers on HIV viral load and DBS specimen collection, testing, and results tracking.

**Study design:** this descriptive study was conducted in Kigoma region in June 2022, covering January to June 2022. We compared 3 months before the intervention (pre-intervention arm) to 3 months after the interventions (post-intervention arm).

**Study participants:** the participants were any healthcare providers who received the comprehensive package for HIV and AIDS management. These included those who were working among the selected CTCs in Kigoma region in areas of HIV testing in the community, providers-initiated testing and counseling (PITC), antiretroviral dispensing, reproductive and child health (RCH) clinics, and laboratories.

**Sampling strategy:** we did a purposeful sampling technique focusing on those high-volume CTCs in the region. CTCs were enlisted with the current number of people living with HIV, and from the list, the top 27 CTCs were included in the assessment to have a representative sample.

**Data collection tool:** under close guidance from the NACP of the Ministry of Health Tanzania, we selected indicators from the DHIS2 database focusing on the underperforming indicators before implementing the comprehensive interventions. Then, we reviewed and contextualized the NACP-approved assessment questionnaires by rephrasing and omitting the questions which were found irrelevant to Kigoma region context, including HIV and AIDS service in the refugee population. The questionnaire was piloted at Kigoma Region Referral Hospital for validity and consistency.

**Variables definition:** assessment variables were the number of new people living with HIV (PLHIV) who tested positive for HIV in a respective month. Sexual partners who were mentioned to have a sexual relationship with PLHIV consented to test for their HIV status. Other variables were PLHIV eligible for HVL specimen collection, HVL specimen results returned, and PLHIV who were eligible for ARV Multi-Month Dispensing (MMD). New people living with HIV who were asked to mention their sexual partners (index client elicitation), the number of HIV-positive index clients (yield), HIV viral load (HVL) specimen, the number of HVL results suppressed, and PLHIV given ARV for more than one month (Multi-month dispensing) (Table 1).

**Table 1:** elaboration of how the study variables were measured against assessed indicators in Kigoma region from January to June 2022

Study theme	Indicator	Study variable	How the variable was measured
Elicitation of newly diagnosed people living with HIV	Number of new people living with HIV	New people living with HIV elicited	We assessed this indicator by the total number of new HIV-positive individuals who were elicited to mention their sexual partners in the month against the new HIV-positive in the respective month. The proportion of elicitation among newly diagnosed HIV-positive was looked at an interval of 6 months; 3 months prior and three months post-interventions.
Mentioned sexual partners during people living with HIV and AIDS (PLHIV) elicitation	Sexual partners consented to HIV testing	Number of HIV positive among sexual partners (percentage yield)	We assessed this indicator by the total number of HIV positive among sexual partners (elicitation yield) against sexual partners mentioned during elicitation and consented to test for their HIV status. Proportion yields were calculated in each respective month and we compared the yields before and post-interventions.
Antiretroviral initiation	New PLHIV eligible for AR initiation	New PLHIV initiated on antiretroviral treatment (ART)	We assessed the indicator by considering a total of newly diagnosed HIV-positive clients against those who were initiated on ARV. We calculated the percentage of initiation in each month of the study
Antiretroviral initiation	Number of pregnant Women living with HIV	Number of pregnant women living with HIV initiated on ARV	We assessed this indicator by looking at the number of pregnant women living with HIV in each month of the study compared with those who were kept on ARV.
Antiretroviral therapy initiation and multi-month dispensing	PLHIV eligible for multi months dispensing (MMD)	PLHIV given MMD	The Multi-Month Dispensing (MMD) was assessed by the proportion of eligible people living with HIV (stable clients) against those who were given MMD dispensing (provision of ART doses to cover more than one visit).
Proportions of viral load sample collection	People living with HIV are eligible for sample collection	HVL samples collected	We assessed this indicator by the proportion of HIV Viral Load (HVL) specimens collected in consideration of PLHIV who were eligible for sample collection, in which the pre and post interventions percentages were calculated.
HIV viral load suppression status among people living with HIV	HVL results returned	Percent of people living with HIV whose HIV viral load was suppressed	We measured the indicator considering the number of PLHIV whose viral load samples were collected and the HVL was found to be high despite being on regular and consistent Antiretroviral treatment. The percentages average for respective months were calculated.

**Data collection:** the assessing team comprised five technical officers, including Regional AIDS Control Coordinator, Regional Data Manager, Regional CTC Coordinator, Regional Quality Improvement focal person, and Public Health Officer from the WHO Kigoma field office. The team reviewed National AIDS Control Program assessment checklist and contextualized it to fit in Kigoma context. The assessment checklist was pre-tested to familiarize with the checklist and validation of the data to be collected. Upon reaching respective CTCs, we reviewed both community-based HIV and AIDS services registers, including HIV testing services registers and index client elicitation registers. We further reviewed ARV registers, isoniazid preventive therapy (IPT) registers, Outpatient department attendance registers), Provider Initiated Testing and Counselling (PITC) registers, PMTCT registers, ART dispensing, and HIV Viral Load registers. For the computerized CTCs, we reviewed the care and treatment system used to record PLHIV information at a health facility level (CTC2 database).

**Data management and analysis:** the collected information was entered into an MS Excel sheet for data cleaning omitting incomplete data, erroneous information, mismatching data, and indicators of which the data were not collected during data collection. Then, we transferred the clean datasheet into STATA software version 13 (Stata Corp, Texas USA) for analysis. Monthly data and respective month proportions were calculated, the percentage change of the indicators was calculated, and the results were summarized in tabular forms.

**Ethical consideration and confidentiality:** the assessment was a routine after-health intervention assessment organized by Kigoma Health Management Team and approved by Assistant Region Administrative Secretary for Health. Before the field visit to CTCs, a courtesy visit was conducted to the respective Head of Health Department in the Districts and, subsequently, the health facility in-charges of the CTCs included in the study. The populated questionnaires from respective CTCs were labeled with letters to maintain confidentiality.

## Results

The trend of the proportion of new HIV-diagnosed clients who were asked to mention their sexual partners (i.e. index clients elicitation) changed from 70% in January, 87% in February, and 88% in March. For the pre-intervention period (January-March), the average proportion was 84%, and for the post-intervention period was 97% (Table 2 section A). For the months after the interventions, the trend of change was 96% in April, 98% in May, and 98% in June. During the pre-interventions period, sexual partners mentioned were 101, and post-interventions were 246, with a positive change of 145 individuals. The number of sexual partners who consented to test their HIV status changed from 39 to 109, with a positive change of 70 individuals. The elicitation products and percentage yields among consented sexual partners increased from 9%-January 12%-February, 16%-March, 25% April, 25% May, and 28% June (Table 2 section B). The ARV initiation for newly diagnosed HIV individuals was at high proportions ranging from 85% to 100%. The proportion achievement for the respective month was 100%-January, 100-February, 85%-March, 90% for April, 100%-May, and 100%-June (Table 3 section A).

**Table 2:** the trend of HIV identification and positivity among sexual partners from January to June 2022 in Kigoma

A. HIV identification in comparison to new people living with HIV asked to mention their sexual partners			
Months	Newly diagnosed people living with HIV (n)	New people living with HIV asked to mention their sexual partners	Percent of mention (%)
January	16	11	70
February	31	27	87
March	48	42	88
April	40	38	96
May	88	85	97
June	56	55	98
Total	279	258	92
**Period (quarters)**			
Jan - March	95	80	84
April - June	184	178	97
B. Positivity of new HIV infection among sexual partners who consented to test for their HIV status			
**Months**	**Sexual partners mentioned during new PLHIV elicitation**	**Sexual partners mentioned and consented to test for HIV**	**Number of HIV positive among sexual partners (%)**
January	23	7	1 (9)
February	47	20	3 (12)
March	31	12	2 (16)
April	44	31	5 (25)
May	109	46	3 (25)
June	93	32	2 (28)

**Table 3:** the trend of antiretroviral initiation among newly diagnosed HIV-positive and pregnant women from January to June 2022

(A). Antiretroviral initiation among newly diagnosed HIV positive		
Months	New HIV-positive (n)	New ART initiations [n (%)]
January	9	9 (100)
February	7	7 (100)
March	13	11* (85)
April	19	17* (90)
May	17	18** (100)
June	5	5 (100)
(B). Antiretroviral initiation among pregnant women living with HIV		
**Months**	**New HIV-positive pregnant (n)**	**New HIV positive on treatment [n (%)]**
January	4	3 (75)
February	16	13 (81)
March	6	5 (83)
April	7	5 (83)
May	19	15 (80) ***
June	0	0

* PLHV was found to have TB signs and symptoms, ** One was from previous months, *** Two new PLHIV were not eligible for ART initiation

Among new HIV-positive pregnant women, the ART initiation for the respective month was 75%-January, 81%-February, 83%-March, 83%-April, and 80%-May. There was no available information month of June (Table 3 section B). The trend of Multi-Month Dispensing (MMD) increased throughout the period under the study. January was 89 %, February 92%, March 90%, April 97, and May 99%. The average proportions for pre- and post-interventions were 90% and 98%, respectively, with a proportion change of 8% (Table 4). The percentage of HVL results returned for January was 66%, February was 73%, March 53%, April 76%, May 68%, and June 9%. The turnaround time (TAT) for HIV viral load testing results for 3 months of pre-interventions was 32 days for January, 43 days for February, and 29 days for March. For three months´ post interventions, TAT was 14 days (Table 5 section A). The HIV viral load suppression among people living with HIV for months pre-interventions was 96% for January, 94% for February, and 94% for March. Three months´ post-interventions were 99% for April, 99% for May, and 100% for June (Table 5 section B).

**Table 4:** the trend of multi-month antiretroviral drugs dispensing among eligible people living with HIV from January to May 2022 in Kigoma region

Months	People living with HIV eligible for multi-month dispensing (n)	People living with HIV are offered multi-month dispensing (n)	Percentage of multi-month dispensing (%)
January	1073	951	89
February	979	898	92
March	1209	1088	90
April	1107	1071	97
May	1367	1333	99
Total	5735	5341	93

**Table 5:** the trend of HIV viral load specimen collection, the results turn around to time viral suppression status from January to June 2022 in Kigoma region

A. HIV viral load specimen collection, the results turn around to time				
Months	Eligible for sample collection (n)	Samples collected (n)	Results turned [n [%)]	Average turnaround time (days)
January	3795	3269	2168 (66)	32
February	2748	2896	2113 (73)	43
March	2317	3572	1899 (53)	29
April	2582	2668	2017 (76)	14
May	2772	2798	1907 (68)	14
June	612	601	53 (9)	14
B. HIV viral load suppression status among people living with HIV				
**Months**	**Results turned (n)**	**Viral suppression (n)**	**Percentage of HIV viral suppression (%)**	
January	2168	2074	96	
February	2113	1996	94	
March	1899	1790	94	
April	2017	1994	99	
May	1907	1896	99	
June	53	53	100	

## Discussion

We observed a significant gain in the HIV and AIDS service monitoring indicators as we compared the pre and post interventions arms of the study periods. There was a notable gain in the identification of new HIV-infected individuals, sustainably enrollment into care and treatment with a significant proportion gain in the HIV viral suppression among people who live with HIV infection. The identification of new HIV-infected individuals was compared before implementation and after the implementation of the comprehensive capacity-building package for HIV and AIDS services. We found a proportion gain of 13% in accelerating the Region’s effort of achieving the first 95% of the global target on HIV and AIDS services. A similar finding was found in the study by Phaswana-Mafuya N *et al*. in a rural area of the Eastern Cape, South Africa [11].

During the assessment, we found an exponential increase in newly diagnosed people living with HIV being asked to mention their sexual partners (elicitation) [12]. We further found an increase of 14% in the proportion of new HIV infections among sexual partners who were mentioned during the elicitation of index clients. This finding simulates the finding in the study conducted by Boeke *et al*. 2018 Uganda, which showed an HIV positivity increase in a special group of HIV exposure [13]. However, it contradicts the finding in the study done by Moucheraud C *et al*. conducted in 2022 Malawi, which shows a decrease in new HIV infections among adults at outpatient clinics [14].

On enrollment in CTCs of newly diagnosed people living with HIV, we did not find a notable change in the number of enrolled people living with HIV. However, we noted a significant number of newly diagnosed people living with HIV who presented signs and symptoms suggestive of tuberculosis co-infections implying late presentation to health facilities. A review conducted by Girardi E *et al*. 2017 to sub-saharan Africa papers has found similar findings indicating PLHIV late presentation to CTC and co-infected with other opportunistic infections such as tuberculosis [15]. We also found many pregnant women who were positive for HIV infection at first attendance at RCH clinics, similar to the study by Kharsany *et al*. in 2015 in rural South Africa [16]. In addition, we found many pregnant women living with HIV who were not offered antiretroviral therapy, predisposing their unborn child to the risks of in-utero HIV infections. The UNAIDS gap report 2014 documented similar findings of many pregnant women living with HIV not being offered ARV during pregnancy [17]. The finding contradicts the U.S. Department of Health and Human Services recommendation for sub-African countries [18].

During the assessment, we looked at the trend of antiretroviral drugs’ multi-month dispensing among eligible people living with HIV. We found a change in proportions from 90% to 98% for pre- and post-intervention, respectively. Ruhago G *et al*. 2022 Tanzania found similar findings, indicating a high percentage of MMD distribution to stable PLHIV [19]. To ascertain the HIV viral load suppression status as one of the global targets to be achieved by 2025, we assessed the HIV viral load suppression status among people living with HIV. We found a positive shift in proportions before and post-interventions, which is recommended in the WHO consolidated HIV Strategic information guide 2020 [20]. During the assessment, we found a decrease in turnaround time for HVL test results from an average of 35 days before and 14 days after the interventions. Matovu JK 2021 in Uganda reported on the significance of shorter turnaround time concerning HVL and PLHIV monitoring [21].

Our study had the following limitations: Our study had a short time post interventions period which may have negatively affected the cumulative impact of the interventions. There was a mix of database sources; for facilities with no computerized database posing a question of data accuracy, the data were extracted from service registers, while for facilities with a computerized database, we extracted from a computer database. We further focused on the high-volume care and treatment that may have increased the change proportions.

## Conclusion

The study demonstrated the accelerated achievement in HIV and AIDS service delivery, which was driven by the dissemination of a comprehensive package of HIV and AIDS management. We recommend promoting in-service capacity building in terms of comprehensive training, equipment supply, and infrastructure availability.

## What this study adds

- Programmatic interventions increase the identification of newly HIV-positive individuals in the community;
- Multi-month dispensing among stable PLHIV improves efficiency and sustainable service delivery and improved PLHIV stability status.

### What is known about this topic

- Joint effort and multi-section service delivery approaches need to be collectively implemented to attain the global target of 95 95 95 goals for HIV and AIDS service delivery by the year 2030. Especially when designing interventions that focus on the marginalized and at-risk groups including refugees in the refugee camps.
